# Effectiveness of holistic assessment–based interventions in improving outcomes in adults with multiple long-term conditions and/or frailty: an umbrella review protocol

**DOI:** 10.11124/JBIES-22-00406

**Published:** 2023-05-04

**Authors:** Stella Arakelyan, Nazir Lone, Atul Anand, Nataysia Mikula-Noble, Marcus J Lyall, Luna De Ferrari, Stewart W. Mercer, Bruce Guthrie

**Affiliations:** 1 Advanced Care Research Centre, Centre of Population Health Sciences, Usher Institute, University of Edinburgh, Edinburgh, UK; 2 NHS Lothian, Royal Infirmary of Edinburgh, Edinburgh, UK; 3 Centre for Cardiovascular Science, University of Edinburgh, UK; 4 School of Medicine, The Chancellor's Building, University of Edinburgh, Edinburgh, UK; 5 School of Informatics, Informatics Forum, University of Edinburgh, Edinburgh, UK

**Keywords:** frailty, holistic assessment, multimorbidity, multiple long-term conditions, umbrella review

## Abstract

**Objective:**

This umbrella review will synthesize evidence on the effectiveness of holistic assessment–based interventions in improving health outcomes in adults (aged ≥18) with multiple long-term conditions and/or frailty.

**Introduction:**

Health systems need effective, evidence-based interventions to improve health outcomes for adults with multiple long-term conditions. Holistic assessment–based interventions are effective in older people admitted to hospital (usually called “comprehensive geriatric assessments” in that context); however, the evidence is inconclusive on whether similar interventions are effective in community settings.

**Inclusion criteria:**

We will include systematic reviews examining the effectiveness of community and/or hospital holistic assessment–based interventions in improving health outcomes for community-dwelling and hospitalized adults aged ≥ 18 with multiple long-term conditions and/or frailty.

**Methods:**

The review will follow the JBI methodology for umbrella reviews. MEDLINE, Embase, PsycINFO, CINAHL Plus, Scopus, ASSIA, Cochrane Library, and the TRIP Medical Database will be searched to identify reviews published in English from 2010 till the present. This will be followed by a manual search of reference lists of included reviews to identify additional reviews. Two reviewers will independently screen titles and abstracts against the selection criteria, followed by screening of full texts. Methodological quality will be assessed using the JBI critical appraisal checklist for systematic reviews and research syntheses and data will be extracted using an adapted and piloted JBI data extraction tool. The summary of findings will be presented in tabular format, with narrative descriptions and visual indications. The citation matrix will be generated and the corrected covered area calculated to analyze the overlap in primary studies across the reviews.

**Review registration:**

PROSPERO CRD42022363217

## Introduction

As the global population ages, the burden of multiple long-term conditions (MLTCs) is also on the rise.^1–5^ An estimated 42% (95% CI 38.9%–46.0%) of the global adult population has MLTCs, with no significant difference in prevalence rates observed between low- or middle-income (36.8%) and high-income countries (44.3%).^2^ In the US, around 32.9% of adults report receiving treatment for ≥2 long-term conditions annually, with 20.7% having ≥3, and 12.3% having ≥4 long-term conditions.^3^ The prevalence rates in the UK are around 23% to 27%, with higher rates observed among the elderly and the less affluent.^4–6^ Over 60% of older adults (aged >65) in the UK are affected by MLTCs,^5,7^ with predictions suggesting a doubling of rates of older people with ≥4 long-term conditions by 2035.^8^

MLTCs are associated with functional declines and contribute to frailty.^9,10^ Frailty is an age-related progressive decline in physiological reserves and functions across multiple organ systems, leading to a vulnerable state of health due to poor homeostatic resources.^11^ An estimated 72% of people with frailty have MLTCs, and 16% of people with MLTCs are also frail.^9^ Frailty is associated with decreased resistance to stressors, resulting in rapid changes in health status following a minor event. Frailty-related health deterioration may lead to the development of comorbidities and MLTCs.^9,10^

People with MLTCs and/or frailty are at increased risk of adverse events, including unscheduled hospital admissions, adverse drug events, and premature death.^1^ This is, in part, because people with MLTCs and/or frailty require access to comprehensive care, but often experience single disease-oriented, fragmented, and poorly coordinated care.^12^ They often require complex treatments resulting in polypharmacy, which puts them at risk of adverse drug events.^13^ They often attend multiple appointments, self-manage their conditions, and adhere to lifestyle changes, resulting in a treatment burden. Given that the presence of MLTCs is socially patterned, the effects are worse in adults from disadvantaged communities among whom earlier onset, more complex needs,^14^ and higher treatment burden^15^ are observed. The experiences and care needs of people with MLTCs are heterogeneous, which adds to the challenges of providing effective care.

MLTCs are one of the major challenges facing health services.^1,13^ Health systems urgently need evidence-based, effective interventions to improve health outcomes (eg, quality of life; physical, mental and cognitive functions; outpatient and inpatient services utilization rate; treatment burden) for people with MLTCs and/or frailty who need additional support services.^12,13,16^ Holistic assessment–based interventions (HABIs), which consider individuals’ health, functional, and social conditions, followed by the formulation of personalized care and follow-up,^17^ are viewed as a promising model of care provision for this population.^4^ Hospital HABIs are commonly used in geriatric practice with frail older adults,^4,18^ referred to as comprehensive geriatric assessments (CGAs).^19^ A CGA is a form of integrated care delivered by a multidisciplinary team based on the holistic assessment of older people’s unique needs in terms of function, cognition, depression, nutrition, and medication use.^19^ A Cochrane review on the effectiveness of hospital CGAs found that initiating a CGA upon hospital admission increases the likelihood of older adults returning home compared with those receiving standard care.^20^ The UK NICE guidance on the management of MLTCs (2016)^4^ suggests that low-intensity, community HABIs are effective in improving health outcomes in older adults (aged>65) with MLTCs and frailty. A recent systematic review by Sum *et al.* ^18^ found evidence of the effectiveness of CGAs in improving functional status, frailty, fall, and mental health outcomes, as well as self-rated health and quality of life in community-dwelling older adults (aged ≥75). The effectiveness of community HABIs in improving patient-centered health outcomes and reducing the risk of adverse events in adults (aged ≥18) with MLTCs is unclear.

A systematic review by Smith *et al.* ^16^ found that community interventions led by multidisciplinary teams and targeted at better care coordination, self-management support, and medicine review have the potential to improve experiences of care and health behaviors in older people with MLTCs. However, there is no conclusive evidence that these interventions are effective in improving quality of life and mental health or in reducing health care utilization rates. For example, a phase 3 randomized control trial (the 3D Study) incorporating patient-centered strategies that reflect international consensus on optimal management of MLTCs, found positive effects on patients’ experience of, and satisfaction with, care. At 15 months of follow-up, however, no effects were observed in relation to the primary outcome of quality of life, or on mental health, polypharmacy, and mortality.^21^ In contrast, a phase 2 randomized control trial (the CARE Plus Study), targeting adults with MLTCs from deprived communities, found some evidence of the benefits of a whole-system, primary-care complex intervention in improving patients’ well-being and quality of life. This intervention included longer GP consultations to allow for structured holistic assessment, relational continuity, practitioner training and support, and patient self-management support.^22^ A Cochrane review evaluating community interventions for people with MLTCs established no clear evidence of benefit in clinical outcomes^23^; however, the included studies had to be targeted at people with MLTCs. This means that potentially relevant interventions from other disciplines using different terminology (including literature on CGAs) were not included.

Recent reviews highlight that uncertainties remain about effective models of care and interventions for adults (aged≥18) with MLTCs,^16,23^ calling for further research into complex interventions prioritizing patient-identified needs and outcomes. The NICE guidelines specifically called for research evaluating the effectiveness of “holistic assessment and intervention,”^(p.19)^ reflecting that this is often a core component of complex interventions in this field but with variations in implementation modalities and other elements included.^13^ Further, interventions targeting people with MLTCs with very similar components (eg, multidisciplinary review with a whole-person focus) can be included or excluded by reviews based on how they are named. Therefore, this umbrella review aims to comprehensively evaluate the evidence-based literature on holistic assessment–based complex interventions targeted at adults with MLTCs and/or frailty. A preliminary search of *JBI Evidence Synthesis*, the Cochrane Database, JBI Library, and PROSPERO was conducted, and no current or in-progress umbrella reviews on the topic were identified.

## Review questions

- What is the effectiveness of community HABIs in improving outcomes in adults (aged≥18) with MLTCs and/or frailty?
- What is the effectiveness of hospital HABIs in improving outcomes in adults (aged≥18) with MLTCs and/or frailty?

## Inclusion criteria

### Participants

We will include systematic reviews that focus on community-dwelling and hospitalized adults aged≥18 with MLTCs and/or frailty. MLTCs (or multimorbidity) will be operationalized based on the NICE guideline definition^13^ as the presence of 2 or more long-term health conditions in an individual, including i) physical and mental health conditions; ii) ongoing conditions such as a learning disability; iii) symptom complexes such as frailty or chronic pain; iv) sensory impairments such as sight or hearing loss; and v) alcohol and substance misuse. We will adopt the World Health Organization’s definition of long-term conditions, which are described as persistent “health problems that require ongoing management over a period of years or decades.”^24 (p.11)^ Frailty is not an easily described syndrome, and there is no universal consensus on its operational definition.^11^ Further, tools and assessments of frailty vary in their complexity. Therefore, we will include systematic reviews considering both the phenotype of frailty (weight loss, exhaustion, weakness, low physical activity, slowness) and/or the accumulation of deficits approach (loss in ≥1 domain of human functioning, such as physical, psychological, or social domains), using a multidimensional specific frailty validated scale, measurement, or index. We will exclude reviews that focus on children or young people aged < 18, adults aged ≥18 receiving end-of-life care, adults aged ≥18 who have a single long-term condition, or those focusing on people with a single long-term condition with an interest in comorbidity.

### Interventions

We will include studies that evaluate HABIs in the community (home, primary care, outpatient clinic, care, or nursing home), hospital (acute care, general medicine, and geriatric care) or both settings. A holistic assessment is broadly defined as a multidimensional process based on the assessment of an individual’s medical, psychological, and social needs and functional capabilities in order to develop personalized care and follow-up. Holistic assessment is a complex intervention that responds to all factors relevant to the health or illness of a person.^17^ The terminology used to describe HABIs may differ across disciplines; we will therefore consider reviews describing interventions based on the assessment of needs in 2 or more domains of health, and using alternative terminology to describe holistic interventions. Table 1 presents detailed descriptions of the selection criteria.

**Table 1:** Review selection criteria

Domain	Inclusion criteria	Exclusion criteria
Publication type	Peer-reviewed systematic review publications in English.	Conference proceedings, abstracts, and meta-analyses published in the letter-to-editor format, scoping reviews, narrative reviews or overviews, systematic review protocols, and gray literature.
Publication timeline	Published between January 2010 and September 2022.	Published before 2010.
Population	Community-dwelling or hospitalized adults (aged ≥ 18) with MLTCs and/or frailty.	Children and/or young people (aged < 18) with multimorbidity. People with only 2 or more mental health problems and no physical health condition. People who receive end-of-life or palliative care. People with a single long-term health condition. People with a single long-term condition with an interest in comorbidity (eg, cancer comorbidities).
Intervention	HABI that has ≥2 assessment domains. Assessed domains may include physical health, psychological, or mental health status, functional status, or cognitive status. Terminology for HABI can be explicit or not. Alternative terminology may include *holistic evaluation, consultation*, or *management; comprehensive needs assessment, evaluation*, or *consultation; comprehensive geriatric assessment, evaluation*, or *consultation*.	HABI with <2 assessment domains. Complex interventions not including holistic assessment as a component.
Comparator	Any context-specific, standard, or usual care.	Complementary and/or alternative care (care that falls outside of mainstream health care).
Primary outcomes	Health-related quality of life, physical and/or cognitive function, mortality, unscheduled hospital admission, unscheduled care attendance, care home admission.	Adverse events not associated with health care (eg, air/rail/road traffic injuries, occupational injuries).
Secondary outcomes	Adverse drug events, length of hospital stay (bed days/year), “geriatric syndrome” (eg, frailty, falls, delirium).	
Context	Community setting (community home, primary care, outpatient clinic, care or nursing home). Hospital setting (acute hospital or emergency care, general medicine or geriatric care).	Hospice, end-of-life care settings.
Study designs	Systematic reviews (with or without meta-analyses) reporting on randomized controlled trials, non-randomized controlled trials, controlled before-after studies, interrupted time series studies. Mixed-methods, combined, or integrative systematic reviews (with or without meta-analyses), including randomized controlled trials, non-randomized controlled trials, controlled before-after studies, and interrupted time series studies.	Systematic reviews including only observational study designs not acceptable to Cochrane EPOC (case series, individual case reports, descriptive cross-sectional studies, case-control, and cohort studies) and pharmacological studies. Systematic reviews reporting qualitative meta-synthesis only. Systematic reviews reporting theoretical studies or published opinions only.

EPOC, effective practice and organisation of care; HABI, holistic assessment–based interventions; MLTC, multiple long-term conditions

### Comparators

We will consider reviews reporting on any type of comparator intervention, including context-specific standard, or usual care.

### Outcomes

We will consider systematic reviews reporting on health outcomes important to people with MLTCs^25^ and/or frailty.^26^ Guided by a consensus-based, core set of outcomes for MLTCs (COSmm)^25^ and frailty (FOCUS),^26^ the primary outcomes of interest will be health-related quality of life, physical and cognitive function, mortality, unscheduled hospital admission (times/year), unscheduled care attendance (provider visits/year), and care home admission (yes/no), measured by validated instruments or any clinically meaningful metrics. Secondary outcomes will be adverse drug events, length of hospital stay (bed days/year), and “geriatric syndromes” (eg, frailty, falls, delirium). We will consider reviews reporting on key outcomes of interest assessed using validated measures, including i) health-related quality of life: EuroQol Five-Dimension (EQ-5D); Short Form Health Survey (SF-12 or SF-36); Global quality of life (WHOQOL-BREF); Assessment of Quality of Life (AQoL 8); ii) cognitive function: Mini-Mental State Exam (MMSE); General Practitioner Assessment of Cognition (GPCOG); Memory Impairment Screen (MIS); Mini-Cog^TM^; and iii) physical function: Sheehan Disability Scale (SDS); Sherbrooke Postal Q; Frenchay Activities Index (FAI); Activities of Daily Living (ADL) or Instrumental Activities of Daily Living (IADL) Scales; Barthel’s Index (BI); PROMIS Physical Function. This list is not exhaustive and other validated measures of outcomes will also be considered.

### Types of studies

We define a systematic review as a synthesis of evidence that has a clearly stated set of objectives with pre-defined eligibility criteria for study selection; an explicit, reproducible methodology; a systematic search to identify all studies meeting the eligibility criteria; an assessment of the validity of the findings of the included studies; and a systematic synthesis of the characteristics and findings of the included studies. We will include systematic reviews of various types (eg, integrative systematic reviews, mixed-methods systematic reviews, combined scoping and systematic intervention reviews), with or without meta-analyses, reporting on experimental and quasi-experimental study designs, such as randomized controlled trials, non-randomized controlled trials, controlled before-after studies, and interrupted time series study designs. Based on the Cochrane Effective Practice and Organisation of Care (EPOC) group criteria, these study designs are acceptable for evaluating the effectiveness of organizational interventions. We will exclude systematic reviews that report only on observational study designs (eg, case series, individual case reports, descriptive cross-sectional studies, case-control studies, cohort studies) and pharmacological studies. We will also exclude narrative reviews without a formal systematic search, screening, quality appraisal, extraction, and synthesis of evidence, as well as systematic reviews reporting on qualitative or theoretical studies or published opinions only (see Table 1).

## Methods

This protocol was developed according to the JBI methodology for umbrella reviews,^27^ the reporting guideline for overviews of reviews of health care interventions (PRIOR),^28^ and the Preferred Reporting Items for Systematic Review and Meta-Analysis Protocols (PRISMA-P) guidelines.^29^ The protocol was registered with PROSPERO (CRD42022363217).

### Search strategy

Systematic searches will be performed in MEDLINE (Ovid), Embase (Ovid), PsycINFO (Ovid), CINAHL Plus (EBSCO), Scopus, ASSIA (ProQuest), Cochrane Library, and TRIP Medical Database for peer-reviewed literature published since 2010. The date limit is applied to capture the most recent and relevant intervention reviews, given that MLCTs and integrated holistic care are relatively new concepts in health care. The search strategy will apply subject terms and keywords relating to the target population and intervention. The search terms will be combined with the Scottish Intercollegiate Guidelines Network (SIGN) database-specific filters for systematic reviews, with no language restrictions for the search. An information specialist will be consulted to finalize the search strategy, which will be tailored to each database. A search strategy used in MEDLINE (Ovid) is provided in Appendix I. In addition, we will manually search the reference lists of included reviews for other eligible reviews.

### Study selection

The retrieved records will be imported to EndNote v20.3 (Clarivate Analytics, PA, USA) for de-duplication. The de-duplicated RIS file will be transferred into Covidence (Veritas Health Innovation, Melbourne, Australia) for screening. Two reviewers will independently screen the retrieved records against the inclusion criteria, initially based on the titles and abstracts, followed by full-text screening. At the full-text screening stage, only reviews in English will be included due to lack of resources and to time constraints. Reasons for the exclusion of full-text studies will be recorded. Disagreement between the 2 reviewers will be resolved by discussion or via a third reviewer. The search and screening results will be presented in a PRISMA flow diagram.^29^

### Data collection

We will extract data using an adapted and piloted JBI data extraction tool^27^ (see Appendix II). Data will be extracted on i) systematic review characteristics (title, first author, country, year of publication, objective); ii) included populations (age, gender, number of conditions, definitions, and measures used); iii) search strategy; iv) complex interventions (names/types of interventions, country in which interventions were tested, intervention components, holistic assessment domains [if reported], who led assessments [if reported], type of controls, total sample sizes, number of meta-analyses); v) setting (community, hospital, or both); and vi) analysis, health outcomes (types/measures used), and results. For reviews with no meta-analysis, a summary of the authors’ primary interpretation of findings will be extracted. For meta-analyses, we will extract data on pooled effect sizes (eg, rate ratio, risk ratio, odds ratio for dichotomous data, and mean difference or standardized mean difference for continuous data), as well as the corresponding 95% CIs and *P* values. For integrative systematic reviews, mixed-methods systematic reviews, and combined scoping and systematic intervention reviews reporting on experimental and quasi-experimental study designs, we will extract data on pooled effect sizes, 95% CIs, *P* values, and/or a statement summarizing the authors’ primary interpretation of the results.

Systematic reviews exploring similar topics may have considerable overlap in included primary studies. We will create a citation matrix and calculate the corrected covered area (CCA) index to analyse the overlap in primary studies included in reviews.^30^ Based on the guidance of Hennessy and Johnson (2020),^31^ we will further examine the reasons for overlap based on CCA value (see Appendix III for details). The reviews with complete/near complete overlap will be examined for reasons of high overlap and considered for exclusion; higher quality (eg, Cochrane reviews) and/or most recent reviews (if ratings are similar) will be retained.

### Assessment of methodological quality

Methodological quality will be appraised by 2 reviewers using the JBI critical appraisal checklist for systematic reviews and research syntheses (CACSRRS).^27^ The tool comprises 11 items evaluating: i) clarity of the review question; ii) appropriateness of the inclusion criteria; iii) appropriateness of the search strategy; iv) adequacy of sources and resources used to search for studies; v) appropriateness of appraisal criteria; vi) duplicate conduct of quality appraisal; vii) applications used to minimize errors in data extraction; viii) appropriateness of methods used to combine the studies; ix) assessment of publication bias; x) soundness of recommendations for policy and practice; and xi) appropriateness of proposed new research directions. The items are scored based on the checklist as “Y=met,” “N=not met,” “?=unclear,” and “NA=not applicable.”

The JBI CACSRRS tool is not intended to generate an overall score, and the rating of overall quality may be based on certain criteria being met.^27^ We differentiated items 1–3 and 5–10 as critical domains (see Appendix IV). Rating the confidence of review results will be based on weaknesses in critical domains, ranging from “high” (no or one non-critical weakness), “moderate” (more than one non-critical weakness), “low” (one critical flaw with or without non-critical weaknesses), and “critically low” (more than one critical flaw with or without non-critical weaknesses). The results of the critical appraisal will be reported in a table with an accompanying narrative. All studies will undergo data extraction and synthesis; however, depending on the overall results of the critical appraisal, sensitivity analyses may be performed to test the robustness of our conclusions.

### Data summary

The extracted data will be synthesized manually. The summary of findings will be presented in tabular format, with narrative descriptions and visual indications accompanying the tabulated results. Where possible, analysis will be stratified by setting. We will classify interventions using an existing taxonomy of health interventions (eg, EPOC) and use a stop light visual indicator to summarize the effectiveness of interventions.^27^ We will collate the pooled estimates reported in each meta-analysis, providing narrative synthesis to these findings.

In summarizing findings across the reviews, we will use the Grading of Recommendations, Assessment, Development and Evaluation (GRADE)^32^ principles for an overall assessment of the quality of evidence across the reviews for outcomes of interest.^27^ The quality of evidence for a given outcome will be graded as high, moderate, or low based on the overall quality of the systematic reviews and risk of bias in primary studies as well as consistency of results in relation to an outcome (see Appendix V).

## Funding

This study is funded by the National Institute for Health and Care Research (NIHR) under its Artificial Intelligence for Multiple and Long-Term Conditions Programme (NIHR202639). The views expressed are those of the author and not necessarily those of the NIHR or the Department of Health and Social Care.

## Acknowledgments

Ruth Jenkins, information specialist, for assistance with the search strategy.

## Author contributions

SA, NL, AA, ML, LF, NM, SM, and BG conceptualized the umbrella review. BG, SM, NL, ML, and AA secured funding. SA and BG developed the search strategy. SA and BG developed the first draft of the manuscript. All co-authors contributed to the review and editing of the final manuscript.

## Appendix I: Search strategy

### MEDLINE (Ovid)

Search conducted on September 26, 2022, returning 1909 results.

**Table:**

1. Multimorbidity/
2. Chronic Disease/
3. Comorbidity/
4. (multimorbid* or multi-morbid* or chronic disease$ or comorbid* or co-morbid* or polymorbid* or poly-morbid* or multidisease* or multi-disease* or disease cluster* or multiple long-term condition* or multiple chronic disease$).tw.
5. ((coocur* or co-ocur* or coexist* or co-exist* or multipl* or concord* or discord*) adj3 (disease$ or ill* or care or condition$ or disorder* or health* or symptom* or syndrom*)).tw.
6. or/1-5
7. Frailty/
8. Frail Elderly/
9. Frailty Syndrome/
10. (frail* or frail* syndrome or geriatric* syndrom* or vulnerabil* or function*).tw.
11. or/7-10
12. 6 or 11
13. Adult/
14. Young adult/
15. Middle aged/
16. Aged/
17. (adult* or young adult* or middle aged or old* or elder* or geriatric* or gerontol* or ageing or aged).tw.
18. or/13-17
19. Needs assessment/
20. Geriatric assessment/
21. Risk Assessment/
22. Patient-centered Care/
23. Health Services/
24. health services for the aged/
25. Delivery of Health Care, Integrated/
26. ((holistic or whole or comprehens* or complet*) adj3 (assess* or evaluat* or consult* or manag*)).tw.
27. ((integrat* or co-ordinat* or multidisciplin* or patient-centr* or person-centr*) adj2 (care or service$)).tw.
28. ((geriatric or aged or elderly or old age) adj3 (assess* or evaluat* or consult*)).tw.
29. (team$ adj2 (care or treat* or assess* or consult*)).tw.
30. (multidiscipline* adj3 assess*).tw.
31. or/19-30
32. Meta-Analysis as Topic/
33. meta analy$.tw.
34. metaanaly$.tw.
35. Meta-Analysis/
36. (systematic adj (review$1 or overview$1)).tw.
37. exp Review Literature as Topic/
38. or/32-37
39. cochrane.ab.
40. embase.ab.
41. (psychlit or psyclit).ab.
42. (psychinfo or psycinfo).ab.
43. (cinahl or cinhal).ab.
44. science citation index.ab.
45. bids.ab.
46. cancerlit.ab.
47. or/39-46
48. reference list$.ab.
49. bibliograph$.ab.
50. hand-search$.ab.
51. relevant journals.ab.
52. manual search$.ab.
53. or/48-52
54. selection criteria.ab.
55. data extraction.ab
56. 54 or 55
57. Review/
58. 56 and 57
59. Comment/
60. Letter/
61. Editorial/
62. animal/
63. human/
64. 62 not (62 and 63)
65. or/59-61,64
66. 38 or 47 or 53 or 58
67. 66 not 65
68. 12 and 18 and 31 and 67
69. limit 68 to yr=“2010 -Current”

## Appendix II: Draft data extraction instrument

**Table:**

Systematic review details	
Title	
First author/year	
Country	
Objective	
**Included population**	
Age (mean, SD)	
Gender	
Number of conditions	
Definitions/measures used	
Total number of participants	
**Search details**	
Sources searched	
Range (years) of included studies	
Number of studies included	
Type of studies included	
Country of origin of included studies	
**Complex interventions**	
Names	
Types included in a meta-analysis	
Intervention components	
Holistic assessment domains (if reported)	
Multidisciplinary teams/who led the assessments (if reported)	
Type of controls	
Total sample sizes	
Number of meta-analyses	
Setting/context	
**Quality appraisal**	
**Analysis**	
Methods of analysis	
Outcomes assessed (measures used)	
Results	
Significance/direction	
Heterogeneity	
**Comments**	

## Appendix III: Analysis of the degree of overlap in primary studies

### Step 1: Create citation matrix (CM)

The citation matrix (CM) will allow for assessing the amount of overlap at the review level as opposed to the outcome level. The CM will list all primary studies (*r*=rows) included for each review (*c*=columns). The duplicate rows will be removed to ensure that a primary study appearing across reviews is noted in a line. The first occurrence of a primary study will be defined as an index publication (see Table A).

**Table A:** Citation matrix

	Review 1	Review 2	Review 3
Primary study 1	x		x
Primary study 2	x	x	
Primary study 3	x		x
Primary study 4	x	x	x

### Step 2: Calculate corrected covered area (CCA) across the matrix

The overlap in studies across the matrix will be calculated based on the CCA method^30^ by dividing the frequency of repeated occurrences of the index publication in other reviews by the product of index publications and reviews, reduced by the number of index publications (see below).

$$\mathrm{CCA}\;\left(\mathrm{Corrected}\;\mathrm{Covered}\;\mathrm{Area}\right)=\frac{N-r}{\mathrm{rc}-r}$$

*N* is the number of included publications (irrespective of overlaps) in evidence synthesis (this is the sum of the ticked boxes in the citation matrix); *r* is the number of rows (number of index publications), and *c* is the number of columns (number of reviews).

The degree of overlap across the matrix can vary from 0–5% slight overlap, 6–10% moderate overlap, 11–15% high overlap, to>15% very high overlap. Depending on the CCA value, a decision tree developed by Hennessy and Johnson (2020)^31^ will be used to guide our further steps.

### Step 3: Examine the CM for reviews with complete/near complete overlap

The reviews with complete/near complete overlap will be examined for reasons of high overlap and considered for exclusion; higher quality (eg, Cochrane reviews) and/or most recent reviews (if ratings are similar) will be retained.

## Appendix IV: Quality appraisal instrument

JBI critical appraisal checklist for systematic reviews and research syntheses

**Table:**

Reviewer: Author:		Date: Year: Record Number:			
		Yes	No	Unclear	NA
1	Is the review question clearly and explicitly stated?				
2	Were the inclusion criteria appropriate for the review question?				
3	Was the search strategy appropriate?				
4	Were the sources and resources used to search for studies adequate?				
5	Were the criteria for appraising studies appropriate?				
6	Was critical appraisal conducted by 2 or more reviewers independently?				
7	Were there methods to minimize errors in data extraction?				
8	Were the methods used to combine studies appropriate?				
9	Was the likelihood of publication bias assessed?				
10	Were the recommendations for policy/and or practice supported by the reported data?				
11	Were the specific directives for new research appropriate?				
**Overall confidence in the review results based on weaknesses in critical domains*** High (no or one non-critical weakness) Moderate (more than one non-critical weakness) Low (one critical flaw with or without non-critical weaknesses) Critically low (more than one critical flaw with or without non-critical weaknesses)					

*Critical domains: Items 1–3, 5–10.

## Appendix V: Quality of evidence across systematic reviews for the outcome*

**Table:**

Quality of evidence	Criteria
High-quality evidence	One or more updated (published within the last 3 years), high-quality systematic reviews that are based on at least 2 high-quality primary studies with consistent results.
Moderate-quality evidence	One or more updated (published within the last 3 years) systematic reviews of high or moderate quality, based on at least: 1 high quality primary study 2 primary studies of moderate quality with consistent results.
Low-quality evidence	One or more systematic reviews of variable quality, based on: primary studies of moderate quality inconsistent results in the reviews inconsistent results in primary studies.

* Based on the GRADE principles.
